# Cardiac Troponin T in Healthy Full-Term Infants

**DOI:** 10.1007/s00246-019-02199-9

**Published:** 2019-09-05

**Authors:** Jonna Karlén, Mathias Karlsson, Håkan Eliasson, Anna-Karin Edstedt Bonamy, Cecilia Pegelow Halvorsen

**Affiliations:** 1 Department of Clinical Science and Education, Södersjukhuset, Karolinska Institutet, Sjukhusbacken 10, 118 83 Stockholm, Sweden; 2 Neonatal Unit at Sachs’ Children’s and Youth Hospital, Hjalmar Cederströms gata 14, 118 61 Stockholm, Sweden; 3 Department of Medical Sciences, Biomedical Structure and Function, Uppsala University, 751 85 Uppsala, Sweden; 4 Department of Women’s and Children’s Health, Karolinska Institutet, Widerströmska huset, Tomtebodavägen 18A, 171 77 Stockholm, Sweden; 5 Pediatric Cardiology Department, Astrid Lindgrens Children’s Hospital, Eugeniavägen 23, 171 64 Solna, Sweden; 6 Clinical Epidemiology Division, Karolinska Institutet, Karolinska vägen, 171 76 Stockholm, Sweden

**Keywords:** Newborn, Cardiac troponin T, High-sensitivity cTnT

## Abstract

In this prospective cohort study of healthy full-term infants, we hypothesized that high-sensitivity cardiac troponin T (hs-cTnT) would be elevated in cord blood, compared with adult reference values, and that it would further increase over the first days of age. Cardiac troponin T has been shown to be significantly increased in healthy full-term newborns compared with adult reference values, but there is no established reference range. Most studies of cTnT in newborns have been performed before the introduction of high-sensitivity cTnT (hs-cTnT) assay. We conducted a study including 158 full-term newborns, at Stockholm South General Hospital. High-sensitivity cTnT was analyzed in umbilical cord blood and at 2–5 days of age. Median hs-cTnT (interquartile range) in cord blood was 34(26–44) ng/L; 99th percentile 88 ng/L. Median hs-cTnT at 2–5 days of age was 92(54–158) ng/L; 99th percentile 664 ng/L. We conclude that hs-cTnT is elevated in cord blood in healthy, full-term newborn infants compared with adult reference values, and that it increases significantly during the first days of life. Our findings further underline the need of caution when using hs-cTnT as a measurement of cardiac impact in newborns.

## Introduction

Cardiac troponin T (cTnT) has been reported to be elevated in healthy full-term infants [1, 2]. In the neonatal intensive care unit (NICU), cTnT is most commonly used to measure cardiac stress associated with asphyxia and patent ductus arteriosus [3–5]. The lack of established reference ranges makes it difficult to interpret elevated cTnT values during infancy. If elevated cTnT values in hospitalized infants are incorrectly interpreted as abnormal, it might lead to unnecessary interventions. In adult medicine, cTnT has been used to diagnose myocardial infarction since 20 years. It is recommended to use a high-sensitivity cTnT (hs-cTnT) assay when analyzing cTnT as it enables determining the 99th percentile in healthy subjects, the cut-off of interest in adults with suspected acute coronary syndrome, with better precision [6]. According to the latest European guidelines, hs-cTnT values > 14 ng/L (> 99th percentile) are considered pathological [7].

Troponin is a calcium-regulated inhibitory protein complex consisting of three subunits: troponin C, I, and T. Troponin T facilitates the contraction of myocytes and is a cardiac-specific protein expressed in four different isoforms in the human heart (cTnT 1–4) [8]. It has been shown that different isoforms are expressed differently in fetuses and adults [9]. What causes the transient rise of cTnT in newborn infants is not fully understood. One possible explanation could be the transient hypoxia related to delivery, in combination with the physiological circulatory adaptation starting after birth. The circulatory adaptation continues during the first days of life which might explain the elevated values of cTnT seen several days after delivery. It is not clarified at what time point cTnT reaches its highest levels postnatally nor when it equals adult reference values. Several studies have investigated cTnT values in infants after admission for neonatal care [3–5, 10–24]. Only a limited number of studies have determined cTnT values primarily in healthy full-term infants [1, 2, 25–28]. All but one of these studies have been performed before the introduction of hs-cTnT assays [2] and a considerable proportion of cTnT values were even below the detection limit [1, 25]. Cardiac troponin T has in most cases been determined either in cord blood [1, 2, 25, 26, 28] or in peripheral blood [3, 11–13, 17–19, 21, 29]. Only a few studies have done sequential measurements of cTnT, and primarily in asphyxiated or premature infants [5, 10, 16]. We aimed to investigate hs-cTnT values in cord blood and during the first week of age, in healthy full-term infants born either after spontaneous onset of delivery, or after planned caesarean section (CS). We hypothesized that hs-cTnT would be elevated already in cord blood, compared with the adult upper reference limit, and that it would further increase over the first 2–5 days of age. We finally hypothesized that hs-cTnT values would be higher in infants born after vaginal delivery compared with infants born after planned CS.

## Methods

### Study Design and Setting

This prospective observational cohort study was conducted at the department of obstetrics and gynecology at Stockholm South General Hospital in Sweden. Women admitted to the delivery ward expecting to give birth at term age (37w + 0d–41w + 6d) and without any known preeclampsia or intrauterine growth restriction were eligible for inclusion following informed written consent. For the first study cohort, 60 full-term newborns, all born after planned CS March 1 to July 1, 2016 were included. The second study cohort included 98 full-term newborns born after a spontaneous onset of delivery, February 13 to April 14, 2017.

### Data Collection and Definition

Blood samples were collected from the umbilical cord within 10 min after birth, in a 0.5-mL lithium heparinized tube and transported to the accredited local laboratory for analysis within 2 h. The analysis of cTnT was performed using a hs-cTnT assay (Electrochemiluminescence, Cobas e602, Roche Diagnostics) and measured in ng/L, with a minimum detection limit of 5 ng/L. Since 1965, all newborn infants in Sweden are offered a screening test of metabolic disorders by venous blood sampling as soon as possible after 48 h of age [30]. In included newborns, an extra sample of 0.5 mL venous blood for measurement of hs-cTnT was collected at this time point, using the same method for analysis as for cord blood. If an insufficient amount of blood was obtained at first venipuncture, a second attempt was allowed by the ethical approval, given parental consent. Hemolysis in hs-cTnT samples results in lower values compared with non-hemolytic values [31]. Therefore, if hemolysis was > 1 g/L, the sample was categorized as hemolytic, as recommended by the manufacturer (Roche), and excluded from analyses.

### Perinatal Factors

Gestational age was defined according to early second trimester ultrasound. Apgar score was assessed by the attending midwife. Cord blood pH and cord blood base excess were assessed from the umbilical artery and/or umbilical vein within 10 min after birth. If infants were admitted to NICU after delivery, the indication for admission and all diagnoses at discharge were recorded. Birth weight was measured within hours after birth and recorded in grams. Small for gestational age (SGA) and large for gestational age (LGA) were defined according to reference Swedish growth curves, as a birth weight below − 2 standard deviations (SD) or above + 2 SD for gestational age and sex, respectively [32]. Data on maternal age, body mass index (BMI, kg/m^2^), blood pressure at enrollment at the maternal healthcare unit, parity, and prescribed medications were recorded from the national standardized obstetric charts. These charts contain prospective data collected throughout pregnancy for each woman. Data on nicotine use (smoking and/or snuff) at three different occasions (3 months before enrollment at the maternal health care, at the day of enrollment at the maternal health care, and at gestational week 30–32), and indications for vacuum extraction, planned or acute CS were also recorded from the same charts. Pre-pregnancy hypertension was defined as a blood pressure > 140/90 mm Hg or use of any anti-hypertensive medication.

### Statistical Methods

Data are presented as numbers and proportions (%), mean values with standard deviations or median values with interquartile ranges (25th–75th percentile). As values of hs-cTnT were not normally distributed, continuous data were reported as medians and interquartile ranges. The 2.5th and 97.5th were defined as a reference interval and to compare hs-cTnT values with the adult upper reference limit, 99th percentile values were also presented. The sample size gives a 90% power (5% significance level) to detect a doubling of the reference values, i.e. a mean of hs-cTnT of 30 ng/L in newborns compared with the an upper reference limit of 15 ng/L for adults given a standard deviation of 20. To detect a halving or doubling of hs-cTnT between sample one and two (two-by-two comparison), 40 individuals had to be included. In order to study differences between groups, Chi-squared test was used for proportions and Student’s *t* test was used for continuous variables. To test for normal distribution, we used the Shapiro–Wilk test. For non-normally distributed variables, we used Wilcoxon sign-ranked test and Kruskal–Wallis test as non-parametric significance test. A *p* value of < 0.05 was considered statistically significant. In the initial statistical analyses no exclusions were made; however, to take into account external factors, we performed sensitivity analyses to evaluate any potential effect of maternal age, BMI, maternal medication or hypertension during pregnancy, labor induction, duration of labor, augmentation during delivery, delivery by acute CS or vacuum extraction, birthweight, gestational age, or any respiratory distress. All statistical analyses were performed using STATA/IC 15.0.

## Results

**Fig. 1:**
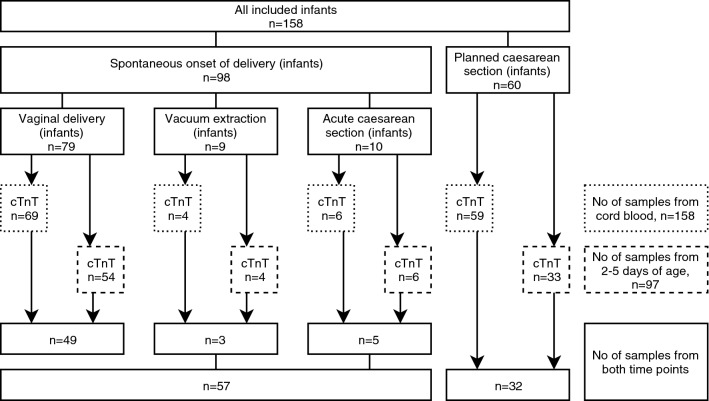
Number of included infants and number of high-sensitivity cardiac troponin T (hs-cTnT) samples according to mode of delivery

**Fig. 2:**
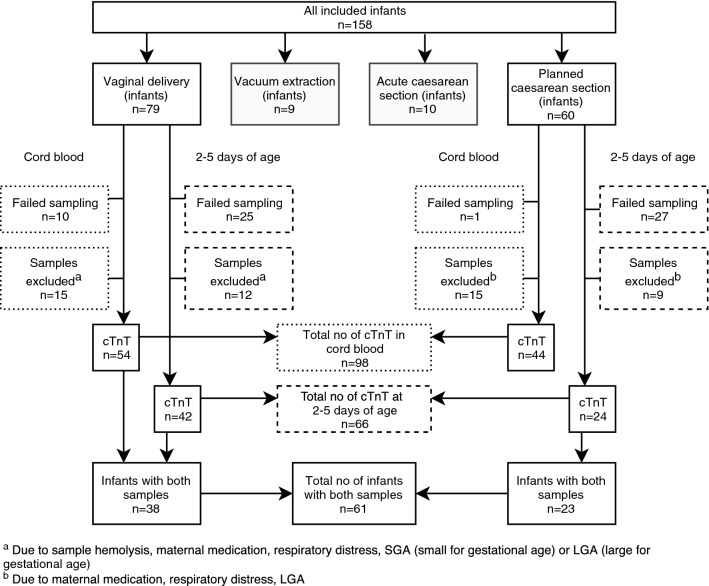
Number of samples of high-sensitivity cardiac troponin T (hs-cTnT) according to mode of delivery, with exclusions

During the study period, 158 newborns were included in the study; 60 were delivered by planned CS and 98 were born after a spontaneous onset of delivery. Indications for planned CS were maternal request (*n* = 35), breech presentation (*n* = 9), previous uterine surgery (*n* = 6), other obstetric cause (*n* = 8), or infant expected to be LGA (*n* = 2). Ninety-eight infants were born after a spontaneous onset of delivery, either by normal vaginal delivery (*n* = 79), acute CS (*n* = 10), or vacuum extraction (*n* = 9). The number of infants and hs-cTnT values according to mode of delivery are shown in Fig. 1. Indication for acute CS or vacuum extraction was risk of fetal asphyxia in 42% of cases. Among spontaneous deliveries, labor was induced in 30 cases (31%) and 63 women (64%) received oxytocin for augmentation of labor. The number of samples of hs-cTnT according to mode of delivery, and exclusions, are shown in Fig. 2. We failed to obtain cord blood samples from 20 newborns due to either an acute event adjacent to delivery (*n* = 1), practical obstacles, (*n* = 2), midwife forgetting to take the sample (*n* = 4), no cord blood sample taken (*n* = 1), or unknown reasons (*n* = 12). At the second blood sampling, at 2–5 days of age, 60 samples were reported missing due to either insufficient amount of blood (*n* = 17), midwife’s choice of not taking the sample (*n* = 5), parents declining a second-sampling attempt (*n* = 7), midwife forgetting to take the sample (*n* = 3), practical obstacles (*n* = 4), or unknown reasons (*n* = 24). Three cord blood samples and three samples taken at 2–5 days of age were hemolytic. In 12 infants, hs-cTnT values were missing in both cord blood and at 2–5 days of age. Seven of them had any respiratory distress. Five of them were born after vaginal delivery, three after acute CS, and four after vacuum extraction.

### Maternal Characteristics

The mean age of all 158 women was 33.2(± 4.9) years and significantly higher in women giving birth by planned CS (34.5[± 4.9] years) compared with those giving birth by vaginal delivery (31.9[± 4.6] years, *p *= 0.001). Maternal characteristics are shown in Table 1. All women had normal blood pressures when enrolled at the maternal health care, but two women had hypertension without proteinuria at the day of delivery and were delivered by acute CS. Approximately 25% (*n* = 40) of the mothers used at least one prescribed medication during pregnancy, the majority stated use of levothyroxine (*n* = 13) or antidepressants (*n* = 9). Twenty-three women stated nicotine use 3 months prior enrollment at maternal health care, five women stated nicotine use at enrollment, and no one stated nicotine use at week 30–32 of pregnancy. There was no difference in proportion of nicotine users between cohorts.

**Table 1:** Maternal characteristics according to mode of delivery

	All included	Planned CS	Vaginal delivery	Spontaneous onset of delivery^a^
	*n *= 158	*n *= 60	*n *= 79	*n *= 98
Age (years)^b^	33.2 ± 4.9	34.5 ± 4.9	31.9 ± 4.6	32.3 ± 4.8
BMI (kg/m^2^)^c^	22.7 (21.4–24.8) *n *= 149	22.7 (21.2–24.8) *n *= 57	22.7 (21.3–24.8) *n *= 74	22.9 (21.4–24.8) *n *= 92
*Parity, n (%)*				
1	71 (45)	20 (33)	37 (47)	51 (52)
2	69 (44)	30 (50)	36 (46)	39 (40)
≥ 3	18 (11)	10 (17)	6 (7)	8 (8)
Any prescribed medication during pregnancy, *n* (%)	40 (25)	21 (35)	14 (18)	19 (19)
At least one prescribed medication at day of delivery, *n* (%)	34 (22)	16 (27)	13 (16)	18 (18)

*BMI* body mass index, *CS* caesarean section

^a^Spontaneous onset of delivery = vaginal birth, vacuum extraction, or acute caesarean section

^b^Mean ± SD

^c^Median (interquartile range)

### Neonatal Characteristics

Median gestational age was 40(39–41) weeks and median birthweight was 3655(3315–3920) g. Detailed neonatal characteristics are shown in Table 2. There were 46% girls and 54% boys. Five infants were born LGA, one infant was born SGA, the rest were appropriate for gestational age (AGA). Median gestational age was significantly lower in infants born after planned CS (39[39–39] weeks) compared with infants born after vaginal delivery (41[40–41] weeks, *p* < 0.001), the same applied to birth weight (3463[3203–3760] vs 3690[3360–3960] g, *p* = 0.03). Median age at second blood sampling was 57(50–81) h. Fifteen infants (9%) had any respiratory distress. All infants had Apgar scores > 7 at 5 min and all infants had normal routine pulse oximetry screening [33] before discharge. Median age at discharge was 2(2–3) days and 92% were discharged as “healthy baby examined at maternity ward” (Table 2).

**Table 2:** Neonatal characteristics according to mode of delivery

	All included	Planned caesarean section	Vaginal delivery	Spontaneous onset of delivery^a^
	*n* = 158	*n* = 60	*n* = 79	*n* = 98
Gestational age (weeks)^b^	40 (39–41)	39 (39–39)	41 (40–41)	41 (39–41)
Birth weight (g)^b^	3655 (3315–3920)	3463 (3203–3760)	3690 (3360–3960)	3725 (3410–4010)
*Sex*				
Female, *n* (%)	72 (46)	30 (50)	37 (47)	42 (43)
Male, *n* (%)	86 (54)	30 (50)	42 (53)	56 (57)
*Apgar score*^*b*^				
1 min	9 (9–9)	9 (9–9)	9 (9–9)	9 (9–9)
5 min	10 (10–10)	10 (10–10)	10 (10–10)	10 (10–10)
10 min	10 (10–10)	10 (10–10)	10 (10–10)	10 (10–10)
Cord pH artery^b^	7.28 (7.22–7.32) *n *= 143	7.31 (7.28–7.33) *n *= 57	7.24 (7.19–7.30) *n *= 69	7.24 (7.19–7.30) *n *= 86
Cord pH vein^b^	7.35 (7.31–7.38) *n *= 144	7.37 (7.34–7.38) *n* = 58	7.33 (7.29–7.37) *n* = 70	7.34 (7.30–7.37) *n *= 86
Any respiratory distress^c^*n* (%)	15 (9)	4 (7)	6 (8)	11 (11)
Age at discharge, days^b^	2 (2–3)	2 (2–2)	2 (1–3)	2 (2–3)
*Discharge diagnosis from maternity ward, n (%)*				
Healthy baby^d^	145 (92)	54 (90)	75 (95)	91 (93)
Transient tachypnea of the newborn	4 (3)	2 (3)	1 (1)	2 (2)
Prenatal hydronephrosis	3 (2)	2 (3)	1 (1)	1 (1)
Ventricular septal defect^e^	1 (1)	0	1 (1)	1 (1)
Other^f^	5 (3)	2 (3)	1 (1)	3 (3)

*CPAP* continuous positive airway pressure

^a^Spontaneous onset of delivery = vaginal birth, vacuum extraction, or acute caesarean section

^b^Median (interquartile range)

^c^Defined as need of assisted ventilation, CPAP within the first hour, diagnosed with transient tachypnea of the newborn or admission to the neonatal ward due to respiratory distress

^d^Infants discharged as “healthy baby examined at maternity ward”

^e^Infant was not sampled at birth or at 2–5 days of age

^f^Skin rash, obstetrical brachial plexus palsy, subgaleal hematoma, congenital dislocation of the hip

### Cardiac Troponin T Analyses

**Fig. 3:**
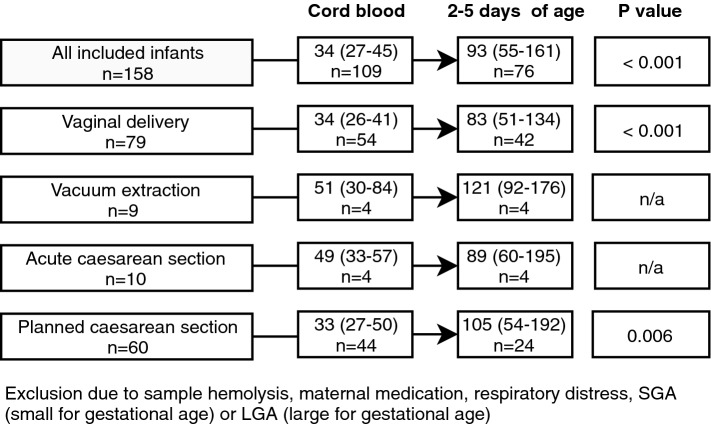
Median (interquartile range) high-sensitivity cardiac troponin T (hs-cTnT) values (ng/L) according to mode of delivery, and difference between first and second blood sample, after exclusion

All hs-cTnT values presented are those after excluding infants with any respiratory distress, infants who were LGA/SGA and infants of mothers with hypertension at delivery or who stated use of antidepressants, antiepileptic medication, oral corticoids, immunosuppressive medication, or antithyroid medication. All hemolytic samples were also excluded (Fig. 2). In this way, only hs-cTnT values in healthy full-term infants born to healthy mothers are reported. High-sensitivity cTnT values are presented in all infants and also separately in infants born after planned CS, infants born after vaginal delivery, and infants born after a spontaneous onset of delivery (vaginal delivery, acute CS, and vacuum extraction). In Table 3, median hs-cTnT values with interquartile range and the 2.5th, 97.5th, and 99th percentile are presented according to mode of delivery. There was no statistically significant difference between hs-cTnT values in cord blood nor at 2–5 days of age when comparing mode of delivery. A statistically significant increase in hs-cTnT values was seen when comparing cord blood samples with samples taken at 2–5 days of age in infants born after vaginal delivery and planned CS. In Fig. 3, differences in hs-cTnT values between first and second blood sampling according to mode of delivery are shown. When analyzing hs-cTnT in all infants (irrespective of mode of delivery), male infants had significantly higher values in cord blood than female infants (39[30–51] ng/L vs 28[24–39] ng/L, *p* = 0.001). However, in samples taken at 2–5 days of age the significant difference disappeared (96[62–157] ng/L vs 85[47–158] ng/L, *p* = 0.40). Values of hs-cTnT according to sex and mode of delivery are shown in Table 4. No significant difference in hs-cTnT was seen with respect to maternal age, maternal use of medication, BMI, labor induction, augmentation of labor, duration of labor, gestational age, birthweight, cord blood pH, or when comparing infants with and without respiratory distress.

**Table 3:** High sensitivity cardiac troponin T (hs-cTnT) values (ng/L) according to mode of delivery, after exclusion

	*n*	Median	2.5th percentile	25th percentile	75th percentile	97.5th percentile	99th percentile
*All infants*							
Cord blood	105	34	16	26	44	76	88
2–5 days of age	73	92	24	54	158	519	664
*Planned CS*							
Cord blood	44	33	22	27	50	76	88
2–5 days of age	24	105	32	54	192	452	452
*Vaginal delivery*							
Cord blood	54	34	16	26	51	64	65
2–5 days of age	42	83	24	51	134	519	664

Exclusion due to sample hemolysis, maternal medication, respiratory distress, small for gestational age (SGA) or large for gestational age (LGA)

**Table 4:** Gender comparison of high-sensitivity cardiac troponin T (hs-cTnT) values in cord blood and at 2–5 days of age, according to mode of delivery

	hs-cTnT ng/L (median, interquartile range)			
	All	Females	Males	*p* value
*All infants*				
Cord blood	34 (26–44) *n* = 105	28 (24–39) *n* = 48	39 (30–51) *n* = 57	0.001*
2–5 days of age	92 (54–158) *n* = 73	85 (47–158) *n* = 33	96 (62–157) *n* = 40	0.40
*Planned caesarean section*				
Cord blood	33 (27–50) *n* = 44	28 (25–33) *n* = 21	41 (30–52) *n* = 23	0.01*
2–5 days of age	105 (54–192) *n* = 24	159 (54–199) *n* = 14	79 (54–150) *n* = 10	0.39
*Spontaneous onset of delivery*				
Cord blood	34 (26–43) *n* = 61	29 (22–40) *n* = 27	38 (30–44) *n* = 34	0.05*
2–5 days of age	85 (58–129) *n* = 49	68 (41–126) *n* = 19	96 (66–162) *n* = 30	0.05*
*Vaginal delivery*				
Cord blood	34 (26–41) *n* = 54	30 (24–40) *n* = 24	35 (28–43) *n* = 30	0.19
2–5 days of age	83 (51–134) *n* = 42	65 (44–107) *n* = 16	96 (65–162) *n* = 26	0.08

*p value ≤ 0.05

Four infants had considerably higher hs-cTnT values at 2–5 days of age compared with the rest of the infants (450–700 ng/L). Three were boys born after vaginal delivery and one was a girl born after planned CS. A third blood sampling was performed in the first two infants who had hs-cTnT values > 450 ng/L (hs-cTnT values in cord blood were missing in both infants). These two infants were boys, born to healthy mothers by vaginal delivery. Both infants had Apgar score 9 at 5 min, normal cord blood gases and were not in need of any respiratory support. The second blood sampling was performed at approximately 4 days of age in both infants. The third blood sampling was performed at eight and 11 days of age, respectively. Values of hs-cTnT were 146 ng/L and 212 ng/L, respectively, thus decreasing but not below the upper reference limit of adults. The infant sampled at day eight, who also had the highest hs-cTnT value at 2–5 days of age, underwent an echocardiographic examination and an electrocardiography, both were considered normal. The assessment was made that there was no indication to carry out any further examination on the infant sampled at day 11.

## Discussion

In this prospective study of healthy infants, term born after planned CS or a spontaneous onset of delivery, we showed that hs-cTnT was elevated already in cord blood compared with adult reference values, and that it increased further during the first 2–5 days of life. The broad range in hs-cTnT seen in our study indicates that even significantly elevated values during this time period might be considered normal. These findings are important, since cTnT and hs-cTnT is used in the NICU to investigate possible cardiac insults due to, e.g., asphyxia, and a misinterpretation of elevated values might result in unnecessary interventions. Our study is the first to do sequential paired measurements of cTnT in healthy full-term newborns using a hs-cTnT assay. Elevated values of hs-cTnT in cord blood, as seen in this study, could indicate that the physiological strain due to delivery leads to cardiac stress. While adapting to the extra uterine life, the newborn undergoes cardiovascular remodeling including closure of the ductus arteriosus, involution of the right ventricle, and significant changes in pulmonary and systemic vascular resistance. This adaptation starting at birth continues during the first days of life and might contribute to the elevated hs-cTnT values seen in infants several days after birth. Previous studies have shown that cTnT is elevated in neonates with respiratory distress [3, 10, 19, 20], in asphyxiated infants [4, 11, 14–16, 18, 22, 24], and in preterm newborns [5]. These studies, as well as other studies investigating normal values in healthy infants, show great diversity in terms of mode of delivery, cTnT assay used, gestational age of included infants (term or preterm), and sampling time point (cord blood or within hours/days) which makes it difficult to compare results. In the largest study to date, cord blood was collected from 869 healthy term newborns and cTnT examined with a third generation assay, which is not a high-sensitivity assay. They reported mean cTnT of 14 ng/L (standard deviations not stated) and a 95th percentile value of 50 ng/L [28]. These values, along with those seen in several other studies, are considerably lower than ours [1, 3, 14, 16, 25, 28, 34]. However, they all used older cTnT assays (generation one, two, or three) that were less precise than the one used in our study. There are a few studies reporting cord blood cTnT values in full-term healthy infants similar to, or higher than ours. A median arterial cTnT of 50(10–50) ng/L was found in 154 newborns born after vaginal delivery [26]. In 57 healthy infants born after acute CS and vaginal delivery, mean cTnT was 33.3(±12.8) ng/L [23]. In 25 infants, also born after acute CS and vaginal delivery, median cTnT was 30(0–42) ng/L [27]. To date, only one published study has reported values of cTnT in healthy full-term newborns using a hs-cTnT assay. Median hs-cTnT-levels in cord blood of 241 infants was 38.2(31.3–48.0) ng/L which is similar to the results seen in our study [2].

Only a few studies have investigated differences in cTnT levels in healthy full-term newborns with respect to mode of delivery, and the results are diverse. Most of them have shown no significant difference in cTnT values (neither in cord blood nor within 48 h of age) when comparing infants born after vaginal delivery and acute CS [23, 27–29]. These findings are consistent with ours. However, a few studies have reported higher values in infants born after vaginal delivery compared with infants born after acute CS [2, 18, 26]. To date, no previous studies have reported cTnT or hs-cTnT values exclusively in healthy infants born after planned CS. We found no significant difference in hs-cTnT values when comparing infants born after planned CS with those born after vaginal delivery. We found this surprising since infants born after planned CS are not thought to be subjected to the same intrapartal transient hypoxia as infants born after vaginal delivery. A limited number of studies have performed sequential measurements of cTnT in premature or asphyxiated infants [5, 10, 16]. Even a fewer number of studies have measured cTnT sequentially in healthy full-term infants [14, 27]. All studies used an older cTnT assay (third generation) than we used in our study. The results are diverse and it is still not clarified how cTnT changes over time postnatally. In our study, hs-cTnT increased significantly during the first days of life, and at 2–5 days of age all infants had values above the upper reference limit used in older children and adults. Further studies are warranted to clarify at what time point hs-cTnT reaches its highest levels after birth and when it equals normal adult values. We found a significant gender-specific difference in cord blood hs-cTnT values in all infants. Male infants had higher values than females. Two other studies have showed similar findings in cord blood and in infants born after a spontaneous onset of delivery [2, 28], whereas several other studies have found no gender-specific differences [1, 3, 12, 18, 20, 26, 27]. Studies with larger sample sizes are needed to investigate this possible association further. We found no significant association between the use of maternal medication and levels of hs-cTnT. However, due to the limited sample size and the relatively small number of women using medication during pregnancy, an effect of maternal medication cannot be excluded. Maternal age or BMI was not associated with hs-cTnT values. Neither labor induction, augmentation of labor with oxytocin, birthweight, nor gestational age were associated with hs-cTnT values in cord blood or at 2–5 days of age. It has been suggested that levels of cTnT in infants might be a useful indicator of myocardial compromise following asphyxia [4, 13]. In our study, hs-cTnT values showed a great range during the first days of life in otherwise healthy infants, and sporadic values were considerably higher than the upper reference limit in adults. These findings underline the need of caution when using single hs-cTnT values as markers of cardiac damage in newborn infants, and even repeated sampling might be of limited use in the clinical setting since the change of hs-cTnT over time is still not fully understood.

### Strengths and Weaknesses

A strength of this study was the inclusion of infants born after planned CS as well as of infants born after a spontaneous vaginal delivery. Inclusion before delivery decreased the risk of selection bias. Detailed, prospectively collected data from maternal health care records made it possible to address important prenatal factors that might affect hs-cTnT levels, such as maternal blood pressure, medication during pregnancy, and BMI. According to our power calculation, we had to include 40 individuals to detect a halving or doubling of hs-cTnT between sample one and two (two-by-two comparison). After exclusion, we had 61 infants with paired samples which enabled a valid estimation of the difference between hs-cTnT values in cord blood and at 2–5 days of age. Most important, no one has to date performed measurements of hs-cTnT in healthy full-term newborns, investigating levels sequentially in cord blood and at 2–5 days of age in the same infant. The main limitation of this study was that we only measured hs-cTnT at two occasions and within a rather narrow time span. We were able to show a significant increase in hs-cTnT values during the first days of life, but we still do not know when hs-cTnT reaches its highest levels after birth or at what time point it descends to normal adult values. Furthermore, due to a limited number of infants it was not feasible to investigate whether there was a significant difference in hs-cTnT values between infants born after vaginal delivery and those born after acute CS or vacuum extraction.

### In Summary

Cardiac biomarkers are used in neonatal care to evaluate potential myocardial compromise due to patent ductus arteriosus or asphyxia. Previous studies have shown elevated values of cTnT in otherwise healthy full-term infants when compared with adult reference values. Our study is the first to do sequential paired measurements using a hs-cTnT assay and we conclude that hs-cTnT is elevated already in cord blood and that it increases during the first days of life. The wide range in hs-cTnT values seen in healthy full-term infants in this study underlines the need of caution when using hs-cTnT values to evaluate cardiac insult in newborn infants.
